# Towards a biological definition of ARDS: are treatable traits the solution?

**DOI:** 10.1186/s40635-022-00435-w

**Published:** 2022-03-11

**Authors:** Lieuwe D. J. Bos, John G. Laffey, Lorraine B. Ware, Nanon F. L. Heijnen, Pratik Sinha, Brijesh Patel, Matthieu Jabaudon, Julie A. Bastarache, Daniel F. McAuley, Charlotte Summers, Carolyn S. Calfee, Manu Shankar-Hari

**Affiliations:** 1 Intensive Care, Amsterdam UMC, Location AMC, 1105AZ Amsterdam, The Netherlands; 2 Anaesthesia and Intensive Care Medicine, Galway University Hospitals, National University of Ireland Galway, Galway, Ireland; 3 Division of Allergy, Pulmonary and Critical Care Medicine, Vanderbilt University Medical Center, Nashville, TN USA; 4 Department of Intensive Care Medicine, Maastricht University Medical Center+, Maastricht, The Netherlands; 5 Department of Anesthesiology, School of Medicine, Washington University, St. Louis, USA; 6 Division of Anaesthetics, Pain Medicine, and Intensive Care, Department of Surgery and Cancer, Imperial College, London, UK; 7 Department of Perioperative Medicine, CHU Clermont-Ferrand, Clermont-Ferrand, France; 8 GReD, Université Clermont Auvergne, CNRS, INSERM, Clermont-Ferrand, France; 9 Wellcome-Wolfson Institute for Experimental Medicine, Queen’s University Belfast, Belfast, Northern Ireland, UK; 10 Department of Medicine, School of Clinical Medicine, University of Cambridge, Cambridge, UK; 11 Division of Pulmonary, Critical Care, Allergy, and Sleep Medicine, Department of Medicine, University of California, San Francisco, San Francisco, CA USA; 12 School of Immunology and Microbial Sciences, King’s College London, London, UK; 13 Centre for Inflammation Research, The University of Edinburgh, Edinburgh, Scotland, UK

**Keywords:** ARDS, Diagnosis, Biomarker, Pathophysiology, Phenotype, Definition

## Abstract

The pathophysiology of acute respiratory distress syndrome (ARDS) includes the accumulation of protein-rich pulmonary edema in the air spaces and interstitial areas of the lung, variable degrees of epithelial injury, variable degrees of endothelial barrier disruption, transmigration of leukocytes, alongside impaired fluid and ion clearance. These pathophysiological features are different between patients contributing to substantial biological heterogeneity. In this context, it is perhaps unsurprising that a wide range of pharmacological interventions targeting these pathophysiological processes have failed to improve patient outcomes. In this manuscript, our goal is to provide a narrative summary of the potential methods to capture the underlying biological heterogeneity of ARDS and discuss how this information could inform future ARDS redefinitions. We discuss what biological tests are available to identify patients with any of the following predominant biological patterns: (1) epithelial and/or endothelial injury, (2) protein rich pulmonary edema and (3) systemic or within lung inflammatory responses.

## Introduction

Acute respiratory distress syndrome (ARDS) was first described in 1967 as a case series. Amongst 272 adult patients receiving respiratory support, twelve patients did not respond to usual management [1]. These twelve patients presented with acute hypoxemic respiratory failure due to non-cardiogenic pulmonary edema with reduced lung compliance and increased work of breathing. The ‘causes’ of acute hypoxemic respiratory failure in these patients included pancreatitis, pneumonia, trauma or aspiration. In 1992, the first consensus definition of ARDS was formalized as the American–European Consensus Conference (AECC) criteria [2], which were updated in 2012 at another consensus conference in Berlin (referred to as the Berlin Definition; Table 1). The concept of acute hypoxemic respiratory failure due to non-cardiogenic pulmonary edema was retained as the ARDS construct within the Berlin Definition [3], with acute defined as within 7 days of insult, and hypoxemia categorised using partial pressure of oxygen/fraction of inspired oxygen concentration ratio (PaO_2_/FiO_2_ ratio), on a positive end-expiratory pressure (PEEP) or equivalent continuous positive airway pressure (CPAP) of 5 cm water. The identified risk factors for ARDS are: pneumonia, aspiration, smoke inhalation, drowning, sepsis, systemic inflammatory response for example in patients with pancreatitis, trauma or surgery, transfusion and toxic medication. These first “hits” are frequently accompanied by a second insult, such as fluid overload, high stress and/or strain on lung tissue or additional blood transfusions.

**Table 1:** Berlin definition

Timing	Within 1 week of a known clinical insult or new or worsening respiratory symptoms	
Chest imaging	Bilateral opacities—not fully explained by effusions, lobar/lung collapse, or nodules	
Origin of edema	Respiratory failure not fully explained by cardiac dysfunction or fluid overload	
Oxygenation	Mild	200 mmHg < PaO_2_/FiO_2_ ≤ 300 mmHg with PEEP/CPAP ≥ 5cmH_2_O
	Moderate	100 mmHg < PaO_2_/FiO_2_ ≤ 200 mmHg with PEEP ≥ 5cmH_2_O
	Severe	PaO_2_/FiO_2_ ≤ 100 mmHg with PEEP ≥ 5cmH_2_O

*PEEP* positive end expiratory pressure, *CPAP* continuous positive airway pressure

Upon histopathological evaluation, patients in the original description of ARDS all had diffuse alveolar damage (DAD) [1]. DAD is the result of a destructive process and injury to all of the alveolar structures is observed. The presence of hyaline membranes (dense eosinophilic amorphous material plastered along the alveolar septa) is one of the hallmark features of DAD, which is frequently seen in combination with white blood cell infiltration, fibrin deposition and collapsed alveoli. Only 45% of patients who fulfilled the Berlin definition of ARDS actually show DAD upon post-mortem histopathological evaluation [4]. ARDS patients without DAD mostly had histopathological features consistent with pneumonia. The introduction of low tidal volume ventilation seemed to decrease the incidence of DAD [4], suggesting that progression towards DAD may not only relate to the disease itself but also reflects ventilation induced lung injury. It is very difficult to predict which patients have DAD based on clinical characteristics alone [5]. It may, therefore, be unreasonable to state that DAD is the histopathological equivalent of what we nowadays consider to be ARDS. Combined with the difficulty of obtaining histopathological samples in patients with ARDS, we will not consider DAD as the reference sample for ARDS in this review (Table 2).

**Table 2:** Summary of biological domains

Domain	Sample material	Example biomarkers	Advantage	Disadvantage
Endothelial injury	Plasma	Ang2	Easy to obtain Pathophysiological contributor to lung injury development	Reflective of all endothelial dysfunction, not only in lung
Epithelial injury	Plasma	sRAGE. SP-D	Easy to obtain Pathophysiological contributor to lung injury development	Not only related to epithelial injury but also to, i.e., clearance by the kidney
Epithelial injury	BALF	sRAGE	Not influenced by clearance Evaluation at site of injury	Difficult to obtain sample Local injury may not be reflective of the rest of the lung
Protein rich pulmonary edema	BALF	Total protein	Direct measurement of hallmark of ARDS	Difficult to obtain sample Local injury may not be reflective of the rest of the lung May not be targetable and reflective of injury to endothelium and epithelium
Protein rich pulmonary edema	EBC	Total protein	Non-invasive collection of EBC Direct measurement of hallmark of ARDS	Requires specialized equipment that is not widely available May not be targetable and reflective of injury to endothelium and epithelium
Protein rich pulmonary edema	HME fluid	Total protein	Non-invasive collection using standard HME Direct measurement of hallmark of ARDS	Novel technique that needs to be validated further May not be targetable and reflective of injury to endothelium and epithelium
Systemic host response	Plasma	IL-6, IL-8, TNFRI	Easy to collect Used to classify subphenotypes	Not unique to ARDS and influenced by other organ dysfunction Unclear contribution to lung injury Not reflective of alveolar inflammation
Alveolar host response	BALF	Neutrophils, macrophages IL-6, IL-8, TNFR1	Direct measurement of hallmark of ARDS Pathophysiological contributor to lung injury development	Difficult to obtain sample Local injury may not be reflective of the rest of the lung

*Ang* angiopoietin, *sRAGE* soluble Receptor for Advanced Glycation End Product, *SP-D* Surfactant protein D, *BALF* broncho-alveolar lavage fluid, *EBC* exhaled breath condensate, *HME* heat moist exchanger, *IL* interleukin, *TNFRI* tumor necrosis factor receptor

**Fig. 1:**
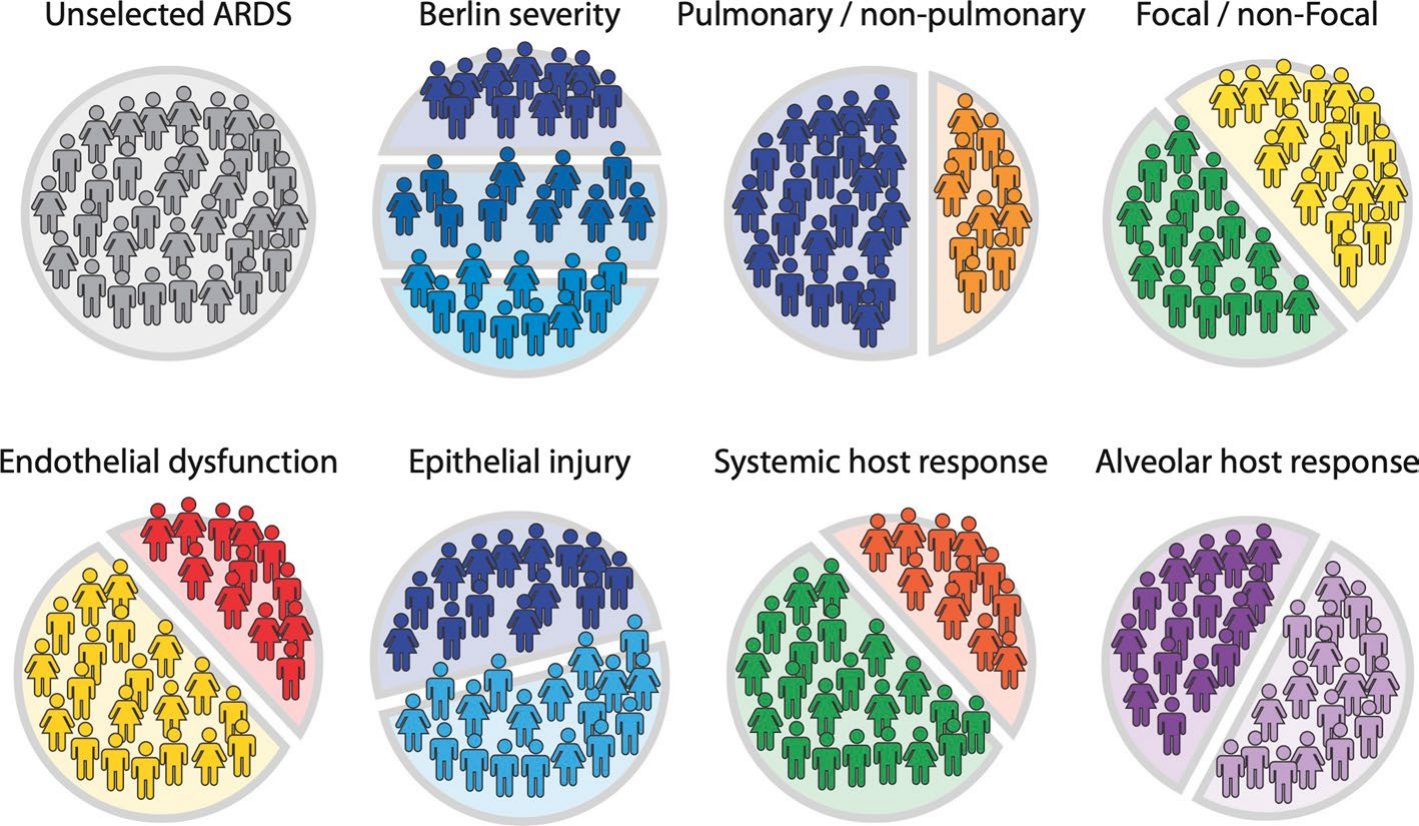
There are many ways to parse ARDS into subgroups. Different ways to parse the ARDS population into subgroups some of which are subphenotypes. One patient can, therefore, belong to many different subgroups simultaneously, each of which could be a treatable trait. Top row from left to right: unselected ARDS; Berlin severity with mild, moderate and severe ARDS based on PaO2/FiO2 (light to dark blue); pulmonary (dark blue) and non-pulmonary (light orange) causes for ARDS; Focal (green) and non-Focal (yellow) ARDS based on chest CT. Bottom row from left to right: patients with (red) and without (yellow) apparent endothelial dysfunction; with (dark blue) and without (light blue) apparent epithelial injury; hyperinflammatory (orange) and hypoinflammatory systemic host response; hyperinflammatory (dark purple) and hypoinflammatory (light purple) alveolar host response

The pathophysiology of ARDS includes the accumulation of protein-rich pulmonary edema in the air spaces and interstitial areas of the lung, variable degrees of lung epithelial injury, variable degrees of endothelial barrier disruption, transmigration of leukocytes, alongside impaired fluid and ion clearance [6]. These pathophysiological features differ between patients, contributing to substantial biological heterogeneity. In this context, it is perhaps unsurprising that a wide range of pharmacological interventions targeting these pathophysiological processes have failed to improve patient outcomes [7, 8]. This biological hetergeneity has another important consequence—it is highly improbable that a single measurement would sufficiently capture the complexity of ARDS to serve as a definitive and reliable diagnostic marker. Therefore, an approach to incorporate relevant biology into the current definition of ARDS would be to assess the relative impact of the main contributing pathophysiological components of ARDS, namely, endothelial barrier disruption, epithelial injury, and both systemic and within lung inflammatory responses. Specifically, in this manuscript, our goal is to provide a narrative summary of the potential methods to capture the underlying biological heterogeneity of ARDS and discuss how this information could inform future ARDS redefinitions. We will also discuss the logistical and technical challenges of using biological diagnoses in the clinical setting. While there are no guarantees that a biologically cognizant definition of ARDS will lead to better therapies, it does seem intuitive that identifying more biologically uniform subgroups may make it easier to identify modifiable targets. Finally, in line with the most prominent pathophysiological changes seen in ARDS, we discuss the biological tests that are available to identify patients with any of the following predominant biological patterns: (1) lung epithelial and/or endothelial injury, (2) protein rich pulmonary edema and (3) systemic or within lung inflammatory responses (Fig. 1).

## Epithelial and endothelial injury

Damage to the alveolar–capillary membrane, which is composed of endothelial, interstitial, and epithelial components, allows for protein-rich pulmonary edema to accumulate in the airspaces of the lung. Measurements of specific biological protein markers in plasma can be used to assess lung epithelial injury (such as surfactant protein-D (SP-D) [9], club cell secretory protein (CC-16) and soluble receptor for advanced glycation end-products (sRAGE) [10]), or endothelial injury (such as angiopoietin-2 (Ang-2) [11] or von Willebrand factor [12]).

Plasma sRAGE is increased in patients with trauma-related ARDS [13] and sepsis-related ARDS [14] and sRAGE has been identified as a promising biomarker for ARDS in several meta-analyses [15, 16]. Increased plasma concentrations of sRAGE and Ang-2 are associated with increased risk for ARDS [11, 17, 18]. While these studies used single biomarkers, others have used panels of biomarkers that reflect the multiple pathophysiological aspects of ARDS. A combination of plasma sRAGE and Ang-2 was superior to clinical assessment for ARDS diagnosis in patients with severe trauma [13], and a panel that included sRAGE, SP-D, and CC-16 was useful for diagnosis in patients with severe sepsis [14, 19]. Elevated plasma concentrations of Ang-2 and sRAGE were positively associated with increased risk of ARDS development, even after multivariable adjustment, in a systematic review of 35 studies involving 10,667 patients at risk for ARDS [16]. More recently, another systematic review of diagnostic methods for ARDS found that plasma CC-16 and sRAGE had good diagnostic accuracy in low-bias studies that compared patients with ARDS to an unselected population of critically ill patients [15]. Higher plasma sRAGE concentrations correlate with impaired alveolar fluid clearance and the severity of lung epithelial injury [20–22], and vary with response to therapeutic interventions in patients with ARDS [23, 24].

sRAGE has been identified as a potential causal intermediate conferring risk for sepsis-associated ARDS in a Mendelian randomization study [25]. This could imply that sRAGE not only is a biomarker of ARDS but that in specific patients, lung injury is driven by sRAGE itself. Similar observations have been made for ANG2 [26]; in one study, plasma ANG2 was found to mediate 34% of the ARDS risk in patients with a specific mutation in the ANG2 receptor gene. These findings suggest that sRAGE and ANG2 are not merely markers reflective of epithelial and endothelial damage, but are pathophysiological contributors to ARDS, at least in a subgroup of individuals.

Assessing the degree of lung epithelial and endothelial injury may be useful to understand heterogeneity to help identify subphenotypes of ARDS [27, 28]. For example, patients with ARDS due to direct pulmonary causes such as pneumonia or aspiration (direct ARDS) have more severe lung epithelial injury, as reflected by higher plasma levels of SP-D and sRAGE, while patients with ARDS due to extrapulmonary causes (indirect ARDS) have more severe endothelial injury, as assessed by plasma Ang-2 [29]. Subphenotypes of ARDS can also be grouped based on the morphology of lung injury into focal and nonfocal ARDS [30], with higher plasma levels of sRAGE and plasminogen activator inhibitor-1 (a marker of endothelial injury) in nonfocal compared to focal ARDS [31]. These two examples illustrate that epithelial and endothelial injury patterns differ in the context of variation in important clinical features.

## Pulmonary permeability and protein rich pulmonary edema

Direct measurement of alveolar–capillary permeability requires measurement of the transit of fluid and protein from the circulation into the alveoli. Elevated alveolar protein concentrations are an excellent surrogate for increased alveolar capillary barrier permeability [32]. While assays to assess alveolar–capillary barrier permeability, such as total protein, albumin, immunoglobulin G and M, and other inflammatory proteins are straightforward and widely available, sampling the distal airspaces to collect pulmonary edema fluid for analysis is more challenging [33], and is seldom part of the clinical workflow. Other approaches for assessing the distal airspaces, and the barriers to their implementation as a clinical diagnostic tool in ARDS, are discussed in detail below.

### Broncho-alveolar lavage fluid

Broncho-alveolar lavage fluid (BALF) is an important method for sampling the distal airspaces in patients with ARDS. Specifically, BALF has been used for identifying causative pathogens; understanding cytological composition; quantifying markers of inflammation, epithelial and endothelial injury; and evaluating the extent of alveolar capillary barrier dysfunction. More recently, BALF has been studied using high-throughput biological measurement platforms (“omics”) with the aim of better understanding host responses and the lung microbiome [34–38].

BALF from patients with ARDS has a significantly higher BALF / plasma protein ratio compared to that of patients with cardiogenic pulmonary edema [39]. Using non-bronchoscopic, minimally invasive approaches to alveolar fluid acquisition, significantly elevated airspace protein concentrations have been shown in ARDS, when compared to cardiogenic edema fluid [33]. Markers of lung epithelial injury have been studied extensively in BALF. Surfactant proteins are known to be decreased in BALF of patients with ARDS compared to patients with other critical illness [40–42]. sRAGE, a marker of injured alveolar type I cells, is elevated in BALF of patients with ARDS [10].

Two factors have limited the clinical and research use of BALF in ARDS. First, bronchoscopy is an invasive procedure associated with risks, albeit risks that are low in patients with acute respiratory failure [43], but can be followed by hypoxemia due to derecruitment. Second, there are considerable limitations in interpreting protein biomarker measurements in BALF or mini-BALF due to inconsistencies in the dilution of the acquired BALF samples [44, 45] and regional heterogeneity. The procedure of the lavage can itself be a determinant of the findings despite consistent procedures [46]. In the absence of therapeutic benefit or interventions made directly as a consequence of BALF findings, its justification as a routine diagnostic intervention is challenging. Yet, studying fluid from the alveolar space should be integral to understanding the biology of ARDS, given its close proximity to the site of injury.

### Exhaled breath condensate

Exhaled breath condensate (EBC) may be useful for characterizing the airspace [47], but current collection equipment is costly, sample volume is limited, and sample collection is labor-intensive [48]. Several studies have measured biomarkers in EBC from patients with ARDS, but few are directly applicable to alveolar capillary barrier integrity. For example, nitrite concentrations increase linearly with tidal volume [49], and proinflammatory cytokines including TNF and IL-8 are elevated in exhaled breath from patients with ARDS, when compared to healthy volunteers [49]. There are no studies of exhaled breath condensate that specifically measure markers of barrier dysfunction, such as total protein or albumin.

### Heat moisture exchange filter fluid

Another more recent non-invasive approach to sampling the airspace in ARDS is extracting fluid from the heat moisture exchange (HME) filter, an inline disposable hygroscopic bacteriostatic sponge routinely placed between the patient and the ventilator. Two recent studies have shown that fluid collected from HME filters reflects the distal airspace in ARDS. Proteomic analysis of HME fluid and fluid collected from direct aspiration of the airspace (as described above) from patients with ARDS or hydrostatic pulmonary edema showed that the proteomic profile of HME fluid is very similar to directly aspirated alveolar fluid [50]. Importantly, total protein can be measured in HME fluid and is higher in patients with ARDS compared to hydrostatic edema [50, 51]. HME fluid analysis may pave the way for incorporating bedside measures of alveolar capillary barrier dysfunction into the definition of ARDS.

## Systemic and alveolar inflammatory response

ARDS is a multifaceted process, which involves both alveolar and systemic inflammation. Inflammation in ARDS is likely influenced by several factors including etiology, host factors (co-morbidities and genetics), immunomodulation (e.g., steroids), the impact of secondary insults (e.g., ventilator-induced lung injury and nosocomial infection) and many others. ARDS also encompasses intra- and inter-individual heterogeneity with respect to spatial and temporal kinetics, and this heterogeneity and the dynamic clinical phenotype of ARDS has challenged the research community with respect to dissecting the role of inflammation. Hence, conceptual frameworks for inflammatory definitions will also need to consider criteria for sampling site, technique, assay specificity/sensitivity, as well as the longitudinal kinetics of alveolar and systemic inflammatory biomarker measurements. Indeed, such approaches will require rapid high throughout bedside assays to enable real-time mapping of disease progression. The ongoing PHIND trial (ClinicalTrials.gov Identifier: NCT04009330) is testing a point of care plasma assay to identify inflammatory subphenotypes of ARDS. Initial data in the setting of ARDS due to COVID-19 provides proof of concept that bedside patient phenotyping in the critically ill may be feasible [52].

Alveolar concentrations of biomarkers of the pro-inflammatory innate immune response, such as interleukin (IL)-1b, IL-6, IL-8 and tumor necrosis factor (TNF) are increased in patients with ARDS [53]. The same challenges associated with obtaining BALF for analysis of protein rich pulmonary edema discussed above also apply for the analysis of intra-alveolar inflammatory markers [54]. Hence, plasma markers are at present studied most frequently as a convenient surrogate to assess pulmonary inflammation, even though a direct association between singular cytokines in both compartments has not been found [53, 55–60].

Plasma concentrations of pro-inflammatory mediators such IL-6, IL-8, TNF receptor 1 (TNFR1) and protein C have driven the identification of ARDS subphenotypes [61–65]. Statistical models that identify homogeneous subgroups of patients (latent class analysis; LCA, and cluster analysis) have consistently identified two subphenotypes, a hyperinflammatory and a hypoinflammatory subphenotype. The hyperinflammatory subphenotype is associated with increased systemic organ dysfunction (as defined by sequential organ failure score), longer ICU stays, and increased mortality [61–65]. Gene expression profiles from blood leukocytes from patients with a more hyperinflammatory subphenotype are reflective of profound neutrophil activation [66]. These systemic inflammatory subphenotypes showed a differential treatment response to PEEP strategy, fluid management, simvastatin administration, and corticosteroids (in patients with COVID-19-related ARDS) [61–65, 67], highlighting their potential importance in the subclassification of patients with ARDS. However, there are also data suggesting that these subphenotypes are not unique to ARDS and might be more widely applicable to critical illness [68, 69].

## Catch 22

One central problem limiting the wide application and implementation of the above discussed biomarkers is that none of these are measured in routine practice, which limits evaluation in large data sets and, therefore, disqualifies them for inclusion in a consensus definition of ARDS. They are currently not measured, because (1) clinical laboratory testing is unavailable and (2) we do not understand the treatment consequences resulting from measurement of these biomarkers. Before we can assess if they would result in superior treatment choices, we need to define the patient population of interest using these biological tests. Hence the Catch 22 of only using routinely available clinical variables is that they only indirectly reflect the underlying injury processes. While ARDS is defined using these variables because of their availability, they do not capture the underlying pathophysiology and biological heterogeneity of the syndrome. Until we can reconcile these two, we will be hampered in our ability to identify distinct biological subtypes within the clinical syndrome of ARDS.

## Focus on treatable traits

Treatable traits are observable biological abnormalities that can be modified such that outcomes are improved. Considering the challenges discussed above, reaching consensus on a fully biological definition of ARDS may be implausible in the short term. Biological data may, however, advance our understanding and treatment of ARDS without the need to reformulate the consensus criteria for ARDS. Thus, the aim of a biological definition should be that it identifies subsets of patients with homogeneous biological characteristics who respond similarly to specific interventions. Rather than trying to generate a biological ARDS definition, we could persist with the broad ARDS diagnosis, as per the Berlin definition, but identify subsets with similar biological features. The corresponding inclusion criteria of an interventional trial would combine the Berlin definition *and* the biological abnormality of interest (such as increased alveolar capillary barrier permeability). If the intervention were to be beneficial in this subset, this would be considered a *treatable trait* within ARDS. In Fig. 1, we summarise how potential treatable traits can co-exist within subsets of the population and thus are not mutually exclusive.

**Fig. 2:**
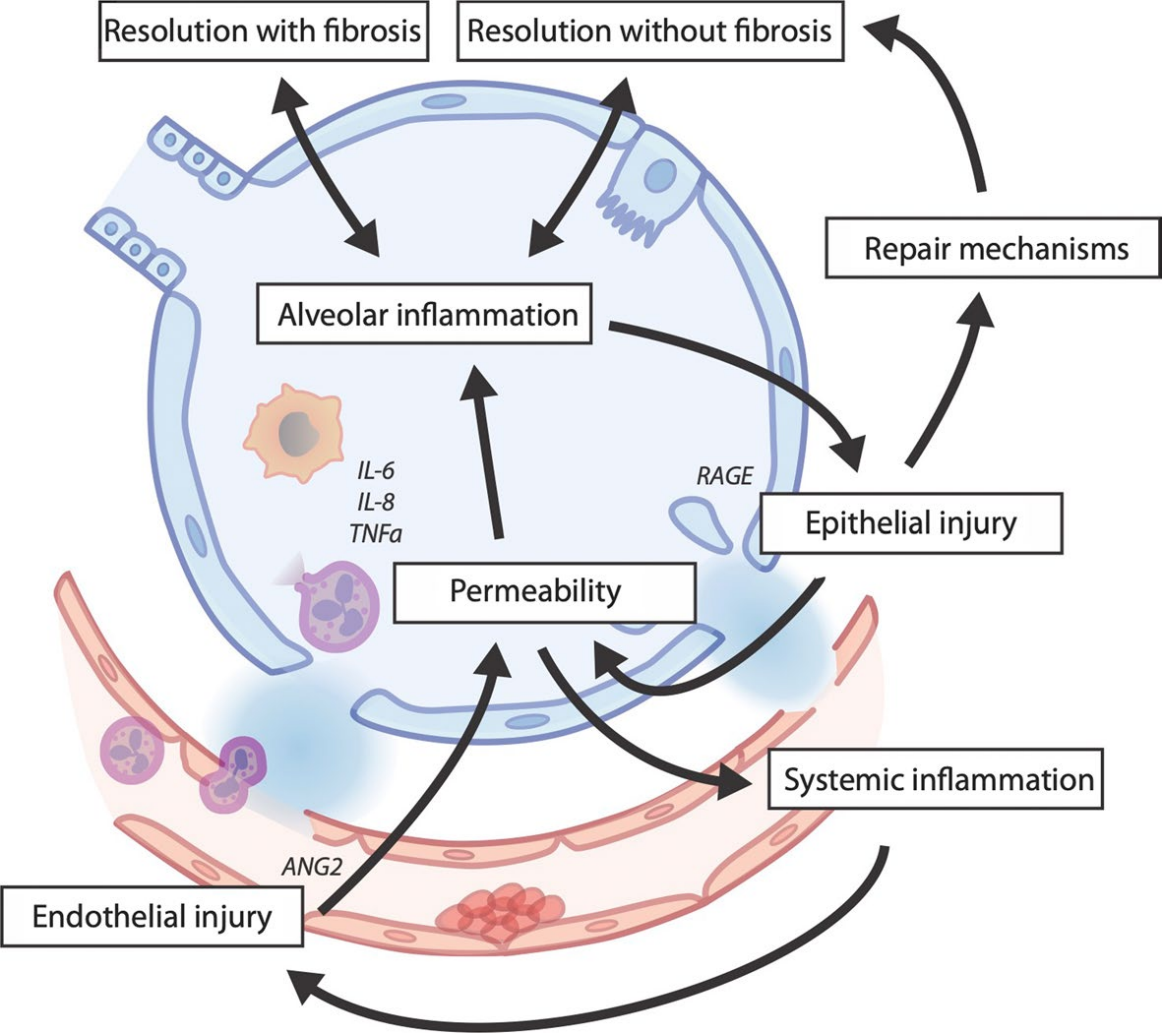
Biological integration of potential treatable traits. The described domains of biological variation do not exist in isolation of each other (Fig. 1). An individual patient could, therefore, be classified according to a conceptional framework that evaluates the three major components of an alveolar unit (endothelium, interstitium with extra-cellular matrix, and epithelium) and the balance of host response between alveolar and blood compartment [71]. We speculate that the position of an individual, based on information pertaining to these component parts in alveolar fluid relative to the circulation, becomes critical in understanding a patient’s biological signature and may inform targeted treatment at a given moment in time. Finally, insights of mechanistic signatures through integration of biological data from other progressive pulmonary pathologies could offer opportunities for drug repurposing in different phases of ARDS, for instance, from interstitial pulmonary fibrosis to ARDS fibrosis [70]

Figure 2 shows how the biological processes that underly potential treatable trait relate. We speculate that the position of an individual, based on information pertaining to these component parts in alveolar fluid relative to the circulation, becomes critical in understanding a patient’s biological signature and may inform targeted treatment at a given moment in time. Finally, insights of mechanistic signatures through integration of biological data from other progressive pulmonary pathologies could offer opportunities for drug repurposing in different phases of ARDS, for instance, from interstitial pulmonary fibrosis to ARDS related fibroproliferation [70].

In this regard, we can learn from the progress made in asthma [72]. Although asthma was considered to be a disease mostly driven by eosinophilic inflammation, the current definition is a syndromic description much like ARDS. Within the most severe forms of ARDS, both neutrophilic and eosinophilic inflammation can be observed resulting in biological heterogeneity. However, the trials of the past decade that showed benefit in asthma only focused on patients with proven eosinophilic inflammation to test antibodies interfering in IL-4 and IL-5 signaling. This treatable trait is now widely recognized and has been included in all guidelines for the treatment of asthma. For ARDS, biological subsets are starting to emerge. When testing an intervention that limits permeability, we could use biomarkers listed in the section on *permeability and edema* to identify patients who are most likely to respond to the intervention. Yet, when testing an anti-inflammatory intervention, we might want to include patients who show evidence for pulmonary or systemic activation of the pathway that is being targeted. Such central alteration of one of the features that leads to lung injury is what we refer to when we discuss a “similar dominant pattern”. This heuristic approach may of course be false: patients with the most activated response are not necessarily the ones who respond most favorably, and these hypotheses need to be verified in prospective randomized controlled trials. At this stage, those trials should generally include both patients who have biomarker evidence of a particular treatable trait *and* those who do not, so as to specifically test whether or not treatment benefits are confined to those with the purported “treatable trait”.

## How to reach the goal of biological treatable traits?

Several steps, outlined below, can be made to bring biological treatable traits closer to reality.

1. Large, inclusive and collaborative biobanks of plasma and alveolar samples from patients with ARDS. Biobanks will need large sample numbers to allow for the identification of subphenotypes, which typically requires hundreds of samples. They ought to be inclusive of the diversity of the patient population experiencing ARDS, because selective sampling would result in biases. Longitudinal sampling can provide additional insights into biological dynamics [73, 74]. To achieve such large and inclusive biobanks, collaborative networks with harmonized collection and processing protocols are needed. Recent NHLBI and ERS workshop reports on Precision Medicine in ARDS made a similar recommendation [28, 75].
2. Biological materials can be used for reverse translational studies, such as in vitro stimulations of alveolar macrophages, neutrophils, endothelial or alveolar epithelial cells. Pharmacological therapies should be tested in such an in vitro setup to inform the pathophysiological changes that can be reversed with this treatment. Subsequently, patients with a similar dominant biological pattern could be selected for participation in intervention studies and the in vitro tests could be used to evaluate intermediate treatment effects in such studies.
3. Biological materials should ideally be collected as part of all RCTs in ARDS patients to allow for testing of heterogeneity of treatment effect in biological subphenotypes.
4. Finally, we should perform intervention studies in cohorts that specifically include patients with ARDS *and* measure the biological factors that enrich the population. Clinical use of such an intervention that only works in a biological subphenotype requires a rapid test for the biomarkers of interest to facilitate inclusion into the trial and time to start treatment and inclusion of selective patients are, therefore, conflicting priorities. Importantly, identifying heterogeneity of treatment effect in post-hoc subgroup analysis of RCT is insufficient and these effects should be confirmed in enriched RCTs.

## Conclusions

To conclude, a true biological definition of ARDS may be out of reach at present due to constraints in data availability and granularity as well as limited understanding of the mechanisms underlying the development of ARDS. With widely inclusive diagnostic criteria, progress could be made through the identification of subgroups with similar biological abnormalities who may have an increased likelihood of responding similarly to specific interventions. This perspective paper provides an overview of the currently available biological data that may be considered in the formulation of such subgroups in a next consensus definition of ARDS. We envision a future where the diagnosis of ARDS is the start of further phenotyping and identification of biological subsets of patients.

## Data Availability

Not applicable.
